# Mental health promotion in the school environment

**DOI:** 10.1093/eurpub/ckac131.498

**Published:** 2022-10-25

**Authors:** R Kouznetsov, E Jelastopulu

**Affiliations:** Department of Public Health, School of Medicine, University of Patras, Patras, Greece

## Abstract

**Issue/problem:**

The practice took place in a private school with students from 9 to 15 years old. The purpose of the intervention was to promote the mental health of students. The school’s goal was to eliminate school bullying incidents. The phenomenon of school bullying has global dimensions.

**Description of the problem:**

The teachers of the school did not consider that they should be involved in issues of mental health of the students and did not take steps to reduce the phenomena of school bullying. In the meetings with the students, which had duration of six months, concepts such as self-esteem, empathy, emotional intelligence, cooperation, respect, stress, failure were analyzed. The causes and consequences of school bullying were discussed. The aim of the meetings was to raise students’ awareness to the above issues, to understand themselves and others and to develop teamwork. In the meetings with the students, three basic principles were developed: Facilitate-Respect-Recognize.

**Results:**

After the end of the practice in the school context, students, teachers and parents completed an evaluation questionnaire anonymously. It turned out that the meetings at school became a cause for discussion in the classroom by 100% and at home by 65%. The students stated that they would not allow school violence or bullying after the meetings. It is noteworthy that one in three teachers did not consider the promotion of mental health as part of their duties while the parents expected the teachers to fulfill this goal 100%.

**Lessons:**

It would be especially useful to conduct training seminars for teachers on adult (to enable better communication channels with parents) and child mental health and conflict management. Respectively, the parents should be educated on issues of mental health and psychopedagogy. The innovation of this action was that it involved teachers, parents and students while similar initiatives seldom take place in a Greek school environment.

**Key messages:**

• Promoting healthy interpersonal relationships at school between students and teachers.

• Recognizing children’s emotions promotes their mental health during school life.

